# Photonic Physical Reservoir Computing with Tunable Relaxation Time Constant

**DOI:** 10.1002/advs.202304804

**Published:** 2023-11-20

**Authors:** Yutaro Yamazaki, Kentaro Kinoshita

**Affiliations:** ^1^ Department of Applied Physics Tokyo University of Science 6–3–1 Niijuku, Katsushika‐ku Tokyo 125–8585 Japan

**Keywords:** photoconductivity, reservoir computing, strontium titanate

## Abstract

Recent years have witnessed a rising demand for edge computing, and there is a need for methods to decrease the computational cost while maintaining a high learning performance when processing information at arbitrary edges. Reservoir computing using physical dynamics has attracted significant attention. However, currently, the timescale of the input signals that can be processed by physical reservoirs is limited by the transient characteristics inherent to the selected physical system. This study used an Sn‐doped In_2_O_3_/Nb‐doped SrTiO_3_ junction to fabricate a memristor that could respond to both electrical and optical stimuli. The results show that the timescale of the transient current response of the device could be controlled over several orders of magnitude simply by applying a small voltage. The computational performance of the device as a physical reservoir is evaluated in an image classification task, demonstrating that the learning accuracy could be optimized by tuning the device to exhibit appropriate transient characteristics according to the timescale of the input signals. These results are expected to provide deeper insights into the photoconductive properties of strontium titanate, as well as support the physical implementation of computing systems.

## Graphical abstract

An SrTiO_3_‐based memristor that could respond to both optical and electrical stimuli is fabricated. This study revealed that the relaxation time of the photo‐induced current could be modulated over two orders of magnitude depending on an applied voltage of less than 0.5 V. Therefore, this device could be applied to physical reservoirs that can process signals over a wide range of timescales with a single device.

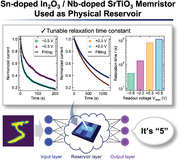

## 1 Introduction

In recent years, the total volume of digital data has increased rapidly owing to the development of networks, devices, and network services such as social media and the cloud. The sophistication and increasing penetration rate of these technologies have introduced a new technological concept called the Internet of Things. We are currently in the big data era, and there has been a dramatic increase in the importance of artificial intelligence (AI) technology to enable the utilization of this enormous amount of data. In conventional computing based on machine learning, a large amount of data is sent to the cloud and processed on the cloud side. This has led to concerns regarding the risk of information leaks and delays due to slow processing speeds. In addition, the data volume increase has enhanced the power consumption. To resolve these issues and improve both the security and processing speed, researchers have focused on edge computing, which operates at the edge regions, that is, in physical proximity to the users and data sources.^[^ ^1^ ^]^ Particularly, AI processing at edge regions, which is called edge AI, is expected to be applied to content that requires real‐time processing where data transfer delays are unacceptable, such as in automated driving systems and machine anomaly prediction in factories.

To enable data processing at arbitrary edges, a machine learning algorithm to reduce the computational cost while maintaining a high learning performance is required. A strong candidate that can meet this requirement is reservoir computing (RC), which is a computational framework suitable for processing time‐series signals. RC transforms these time‐series signals into high‐dimensional spatiotemporal patterns through elements that respond nonlinearly to input signals called reservoirs. RC makes it possible to perform tasks, such as pattern analysis, without using complex algorithms and by learning only when reading out.^[^ ^2^, ^3^ ^]^

Because the role of the “reservoir” in RC is to convert the system input into a higher‐dimensional characteristic vector space, an actual physical system that responds nonlinearly to the input can be used instead. Such a physically implemented reservoir is called a physical reservoir (PR), and the RC that implements a PR (PRC) has been experimentally or computationally demonstrated in various physical systems, including memristors,^[^ ^4^, ^5^, ^6^, ^7^, ^8^ ^]^ magnetic tunnel junctions,^[^ ^9^ ^]^ optical response,^[^ ^10^, ^11^, ^12^ ^]^ soft materials,^[^ ^13^ ^]^ dielectric relaxation at ionic liquid interfaces,^[^ ^14^ ^]^ and the redox reaction of metal ions in ionic liquids.^[^ ^15^ ^]^ However, the timescale of the input signals that can be processed by these PRs, including memristors, is currently limited by the time constant of the transient characteristics inherent to the chosen physical system. To optimize the learning performance of PRCs, the transient characteristics must be appropriately matched to the input signals timescale. Therefore, PR devices with arbitrarily tunable transient characteristics are required for a wide range of edge applications.

Among PRs, memristors^[^ ^16^ ^]^ have the potential for high‐density integration and low‐cost fabrication because of their very simple structure, which consists of an oxide sandwiched between two electrodes (ELs). Even if the PRC attains high performance, hardware implementation becomes difficult if the physical system used for the reservoir is hard to be accepted in semiconductor processes. In this regard, memristors have already been employed in in‐memory computing, which is gaining popularity as a novel architecture to replace Neumann‐type computers,^[^ ^17^, ^18^, ^19^ ^]^ and have a significant advantage over other physical systems in terms of the hardware implementation of RC. Recently, PRC with memristors has been reported to demonstrate high computational performances in tasks such as time‐series prediction, image classification, and spoken‐digit recognition.^[^ ^4^, ^5^, ^6^, ^7^, ^8^ ^]^

In recent years, EL/Nb‐doped SrTiO_3_ (Nb:STO) memristors have attracted attention owing to the continuous, reproducible, and controllable hysteresis of their current–voltage (*I*–*V*) characteristics, which allows them to be applied to a multi‐bit memory as a replacement for flash memory.^[^ ^20^, ^21^, ^22^, ^23^, ^24^, ^25^, ^26^, ^27^ ^]^ In addition, Gao et al. reported their applicability to artificial optoelectronic synapses when the transparent conductor Sn‐doped In_2_O_3_ (ITO) was used as the EL. An ITO/Nb:STO junction was demonstrated for image storage using 3 × 3 cells, and it is expected to be developed for applications that mimic biological functions such as memorizing and learning images captured by visual organs.^[^ ^28^ ^]^

Therefore, this study aims to apply the ITO/Nb:STO junction to a time constant tunable PR device for photo‐learning. Because the electrical conductivity of Nb:STO changes under both optical and electrical stimuli, it was expected that the photo‐induced current characteristics of the device could be electrically controlled. The results of an experiment showed that the relaxation time of the photo‐induced current could be controlled to several orders of magnitude by applying a small bias voltage of less than 0.5 V to the ITO/Nb:STO junction. This result indicated that the ITO/Nb:STO junction is suitable for PR devices and as an AI device that can process various signals in a single device.

## 2 Electrical Characteristics

**Figure 1:**
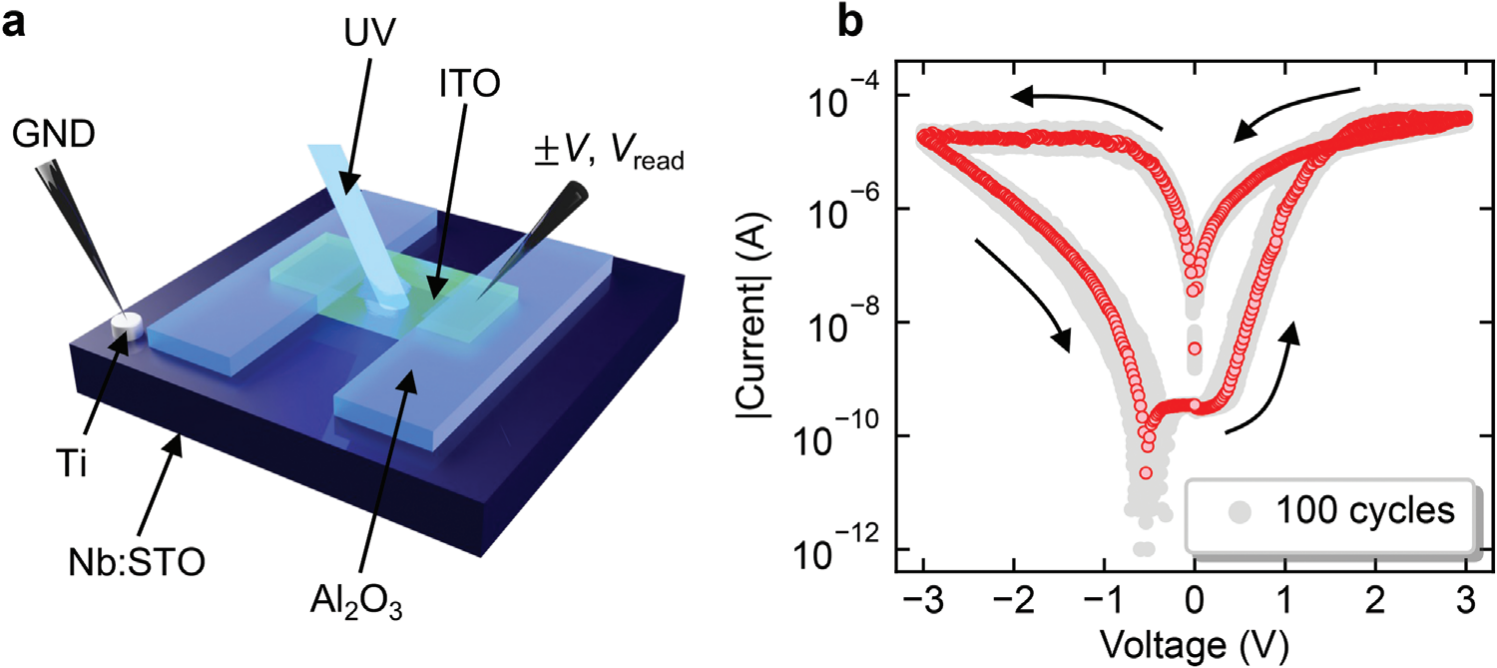
Structure of the photonic memristor and *I*–*V* characteristics: a) schematic of ITO/Nb:STO junction and b) *I*–*V* characteristics of the device. The sweep sequence is marked in the figure. The *I*–*V* curves show the wide memory window and high cycle repeatability.

**Figure** **1**a shows a schematic of the ITO/Nb:STO junction with a structure to avoid the direct contact of the probe with the area of the ITO/Nb:STO interface. Adopting this structure made it possible to prevent light from being reflected by the measurement probe during light irradiation and ensured that the ultraviolet (UV) light was incident on the ITO/Nb:STO interface. See Supporting Information for more details on the device structure (Figure S1, Supporting Information).

Figure 1b shows the *I*–*V* characteristics of the fabricated device, where a voltage was swept repeatedly over 100 cycles, and the following sequence constituted a unit cycle: 0 V → +3 V → 0 V → −3 V → 0 V. Here, the gray and red data show all the *I*–*V* cycles and the typical *I*–*V* cycle, respectively. The resistance was repeatedly decreased and increased through the 100 cycles with high reproducibility by applying positive and negative bias voltages, respectively, indicating that the ITO/Nb:STO junction operated as a memristor. The device was forming‐free, which meant no special treatment was necessary to derive the resistive switching phenomena. See the Supporting Information for the current retention characteristics, cyclic endurance, area dependence of the switching characteristics, and other memristor characteristics (Figures S2– S8, Supporting Information).

Lee et al. reported that the migration of oxygen vacancies (V_O_) caused resistive switching in an Nb:STO‐based memristor.^[^ ^26^ ^]^ The V_O_ acting as dopants migrated from (to) the vicinity of the EL/Nb:STO interface to (from) the bulk Nb:STO side as a result of the application of an electric field. This decreased (increased) the carrier concentration and increased (decreased) the width of the depletion layer, resulting in a resistance increase (decrease). In other words, controllable resistance switching was achieved based on the polarity and amplitude of the bias voltage.

The device characteristics under light irradiation were expected to change significantly with the change in the V_O_ density near the interface resulting from the application of an electric field. This is because STO is an indirect transition semiconductor, and carrier generation and recombination are mediated by defects in the band gap. In the next section, we will discuss how the application of an electric field to the device affected the photo‐induced current characteristics based on experimental results.

## 3 Photo‐Induced Current Characteristics

The photo‐induced current characteristics of the device under the UV irradiation were evaluated. We measured the UV irradiation effect on the time dependence of the current (*I*–*t* characteristics) as a function of a constant readout voltage (*V*_read_). The UV light was emitted from the ITO transparent EL side. The measurement configuration is shown in Figure 1a. The measurements consisted of monitoring the current values of the device for 3000 s, which included a waiting time of 1200 s, UV irradiation for 600 s, and current retention characteristic measurement for 1200 s. The waiting time was set to eliminate the effect of the transient current response immediately after *V*_read_ was applied, which was strongly affected by the capacitance of the Schottky interface.

**Figure 2:**
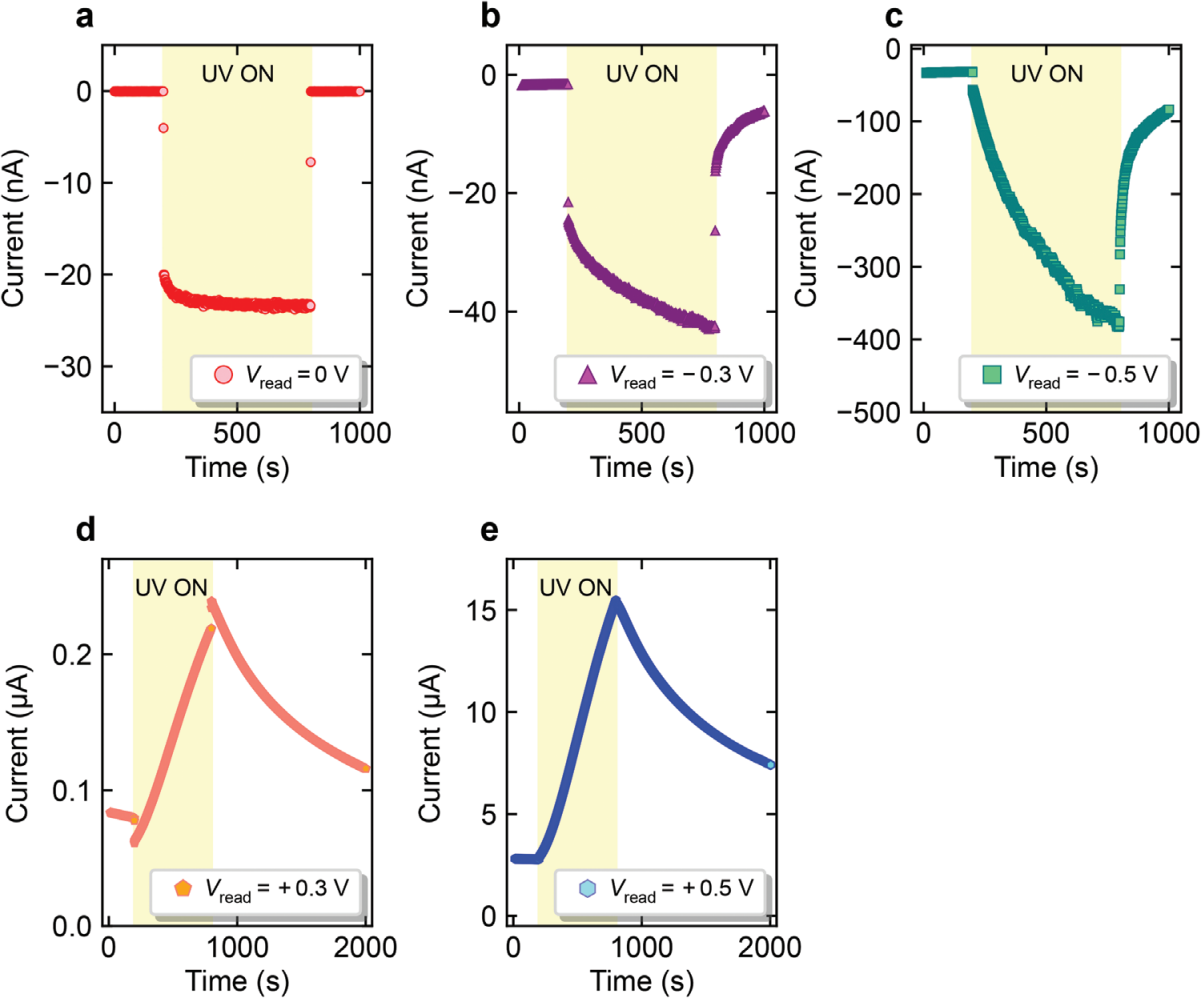
Photo‐induced current characteristics of ITO/Nb:STO junction when *V*_read_ is a) 0 V, b) −0.3 V, c) −0.5 V, d) +0.3 V, and e) +0.5 V.

**Figure** **2**a shows the *I*–*t* characteristics for *V*_read_ = 0 V. A negative current quickly increased to the saturated value just after turning on the UV light and quickly decreased to the original current level before the UV irradiation, where the increase and decrease of the current occurred within less than 1 s. This result was caused by the photovoltaic effect. For *V*_read_ = −0.3 and −0.5 V, the photo‐induced current characteristics of the device changed dramatically, as shown in Figures 2b and 2c, respectively. The photo‐induced current quickly increased immediately after turning on the UV light and, during UV irradiation, the current continued to increase for 600 s without reaching a saturation value. Similarly, after turning off the UV light, the current first decreased rapidly. Then, the current continued to decrease gradually over 250 s without reaching the value before UV irradiation. The amount of changes in current immediately after turning on and off the UV light were similar to those observed at *V*_read_ = 0 when the UV light was turned on and off (20–25 nA). This clearly indicated that applying *V*_read_ drastically changed the transient characteristics of the device compared to the case without applying *V*_read_ (*V*_read_ = 0 V). Figures 2d and 2e show the photo‐induced current characteristics of the device when *V*_read_ was positively biased at +0.3 V and +0.5 V, respectively, indicating a longer photo‐induced current relaxation compared to the negative bias case.

To quantitively evaluate the *V*_read_ dependence of the transient characteristics of the photo‐induced current after the UV light was turned off, the absolute value of the current output by the device was normalized to the maximum photo‐induced current immediately before turning off the UV light and expressed as *I*(*t*) as a function of *t*. The relaxation time was estimated by fitting the extended exponential function^[^ ^29^ ^]^ of Equation (1),

(1) $$\begin{array}{c} I\left(t\right)=A\exp\left[-{\left(\frac{t}{\tau }\right)}^{\beta }\right] \end{array}$$

to the *I*(*t*) data, where *A* is the weight, and τ and β are the relaxation time and expansion/contraction index, respectively. β takes values between 0 and 1, considering the distribution of the relaxation time. **Table** **1** lists the fitting results.

**Table 1:** Fitting results obtained with Equation (1).

*V*_read_ [V]	*A*	τ [s]	β
−0.5	3.1 × 10^−7^	4.2 × 10^1^	0.37
−0.3	1.5 × 10^−8^	1.4 × 10^2^	0.50
+0.3	1.6 × 10^−7^	7.9 × 10^2^	0.87
+0.5	1.3 × 10^−5^	1.1 × 10^3^	0.76

**Figure 3:**
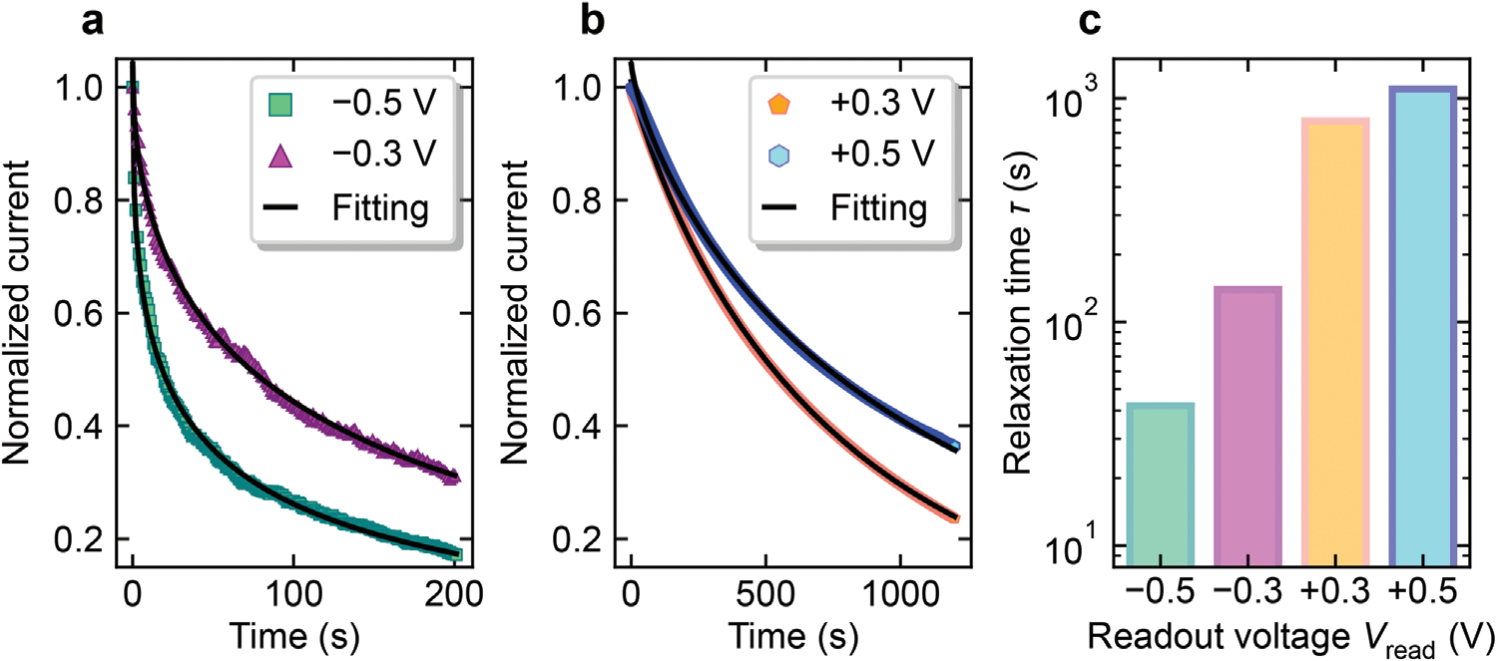
Time dependence of photo‐induced current after turning off the UV light for a) *V*_read_ < 0 and b) *V*_read_ > 0. c) *V*_read_ dependence of the photo‐induced current relaxation time.

**Figure** **3**a,b show the fitting results when *V*_read_ is negatively and positively biased, respectively. When *V*_read_ = 0 V, only the photovoltaic effect was observed, with no current relaxation phenomenon; therefore, it was excluded from the comparison. The *V*_read_ dependence of the relaxation time extracted by fitting is shown in Figure 3c, and it is evident that a small change in *V*_read_ alone could dramatically modulate the device relaxation characteristics.

In general, it is important that the transient dynamics decays with a time constant that is properly matched with the timescale of the input signals to optimize the performance of the PRC. Therefore, an ITO/Nb:STO junction, whose time constant can be electrically controlled through *V*_read_, is expected to be able to optimize the learning accuracy according to the timescale of the input signals. This will be demonstrated in Section 5.

## 4 Working Principle

To investigate defects in the ITO/Nb:STO interface, photoluminescence (PL) measurements at the junction interface were performed. Information regarding these defects in the material could be obtained from the recorded emission spectrum. STOs are indirect transition semiconductors, and the transition of electrons between the conduction band and valence band at the same wavenumber vector is not possible. Therefore, luminescence was observed in oxygen defect‐rich STO surfaces obtained by exposure to Ar^+^^[^ ^30^ ^]^ and high‐energy excitation light,^[^ ^31^ ^]^ suggesting that the mediation of electron transition by defects enhances the electron transition from valence to conduction band and vice versa.

**Figure 4:**
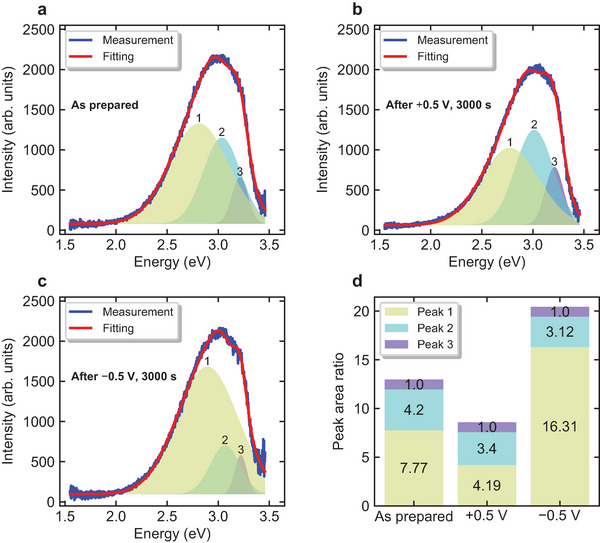
Results of PL measurements under different interface conditions: a) as prepared, b) after applying positive bias, and c) after applying negative bias. d) Area ratio of each peak obtained by peak fitting. Values are normalized by the area of peak 3.

**Figure** **4**a shows the PL measurement results for the ITO/Nb:STO interface without voltage application treatment (as prepared). A broad peak with a peak top at ~3 eV was observed. Such a broad peak is often observed in PL measurements of STO at room temperature.^[^ ^30^, ^31^, ^32^ ^]^ To investigate the recombination centers attributed to the luminescence, we performed peak fitting of the obtained PL spectra. The data were best fitted with the weighted sum of three Gaussian functions. The peaks were located around 2.8, 3.0, and 3.2 eV, and named peaks 1, 2, and 3, respectively. See Supporting Information for the fitting parameters (Tables S1– S3, Supporting Information). This suggested that the energy levels (*E*_T_ values) responsible for the luminescence were located at 0.5, 0.3, and 0.1 eV below the bottom of the conduction band (*E*_C_), respectively, considering the band gap of STO. The *E*_T_ values at 0.5 eV and 0.3 eV below *E*_C_ corresponded to the univalent and divalent oxygen vacancies, V${}_{O}^{+}$ and V${}_{O}^{2+}$, respectively.^[^ ^33^ ^]^ *E*_T_ = 0.1 eV corresponded to Nb with a carrier generation efficiency of almost 100% at room temperature.^[^ ^34^, ^35^ ^]^ Accordingly, peaks 1–3 were energy levels related to V${}_{O}^{+}$, V${}_{O}^{2+}$, and Nb, respectively.

As shown in Section 2, measurements of the photo‐induced current characteristics showed that the current relaxation time of the device changed over several orders of magnitude in response to changes in the applied bias voltage in the range of −0.5 to +0.5 V. We considered the current relaxation phenomenon of the device to be dominated by defects at the interface and performed PL measurements after applying the voltage to clarify the effect of the electric field on the V_O_ (V${}_{O}^{+}$ and V${}_{O}^{2+}$) near the interface. The voltage values and application time were set to +0.5 V (Figure 4b) or −0.5 V (Figure 4c), and 3000 s, respectively, which were the same as the values used during the actual evaluation of the photo‐induced current characteristics. The resistance values after applying +0.5 and −0.5 V for 3000 s were 86 and 135 MΩ, respectively, while the initial resistance was 114 MΩ, which was consistent with the bias polarity dependence in a resistive switching model reported by Lee et al., as discussed in Section 2. See Supporting Information for the *I*–*t* characteristics during the voltage application process used to obtain the PL measurement results (Figures S9 and S10, Supporting Information). Figures 4b and 4c show the PL spectra of the ITO/Nb:STO interface of the device with bias voltages of +0.5 and −0.5 V, respectively. Both spectra could be well fitted using the function applied to the spectrum in Figure 4a, but with different weights for peaks 1–3.

Figure 4d shows the areas of peaks 1–3 normalized by the area of peak 3, assuming that peak 3 was attributed to Nb doping and, thus, unaffected by the application of an electric field. The area of peak 1 decreased (increased) when a positive (negative) bias was applied compared to when a negative (positive) bias was applied. In previous studies,^[^ ^36^, ^37^ ^]^ the relative defect densities were estimated based on the area ratio of the Gaussian functions constituting the PL spectrum. Figure 4d shows that the V_O_ density at the interface decreased (increased) with a positive (negative) voltage application that generated an electric field in the direction from (to) ITO to (from) Nb:STO.

The Shockley–Read–Hall (SRH) model^[^ ^38^ ^]^ is one of the simplest models and expresses the generation and recombination rates of electrons and holes in the presence of energy levels in the band gap of a semiconductor. These energy levels are called recombination centers and mediate the recombination of electrons and holes by capturing/releasing electrons. Based on the SRH model, when light is irradiated to a semiconductor and more carriers are generated than in thermal equilibrium, the recombination rate increases, and the energy level in the band gap acts to reduce the number of carriers. In addition, an increase in the density of the recombination centers increases the recombination rate. The present work showed that the energy levels working as dominant recombination centers corresponded to the V${}_{O}^{+}$ levels, with the density proportional to the area of peak 1 in the PL spectrum. An observation of the photo‐induced current characteristics of the device (Figure 3) suggests that the relaxation speed increased (decreased) because the electronic transitions are enhanced (suppressed) due to the increase (decrease) in the V_O_ density by the application of a negative (positive) *V*_read_.

## 5 Application to Physical Reservoir Device

**Figure 5:**
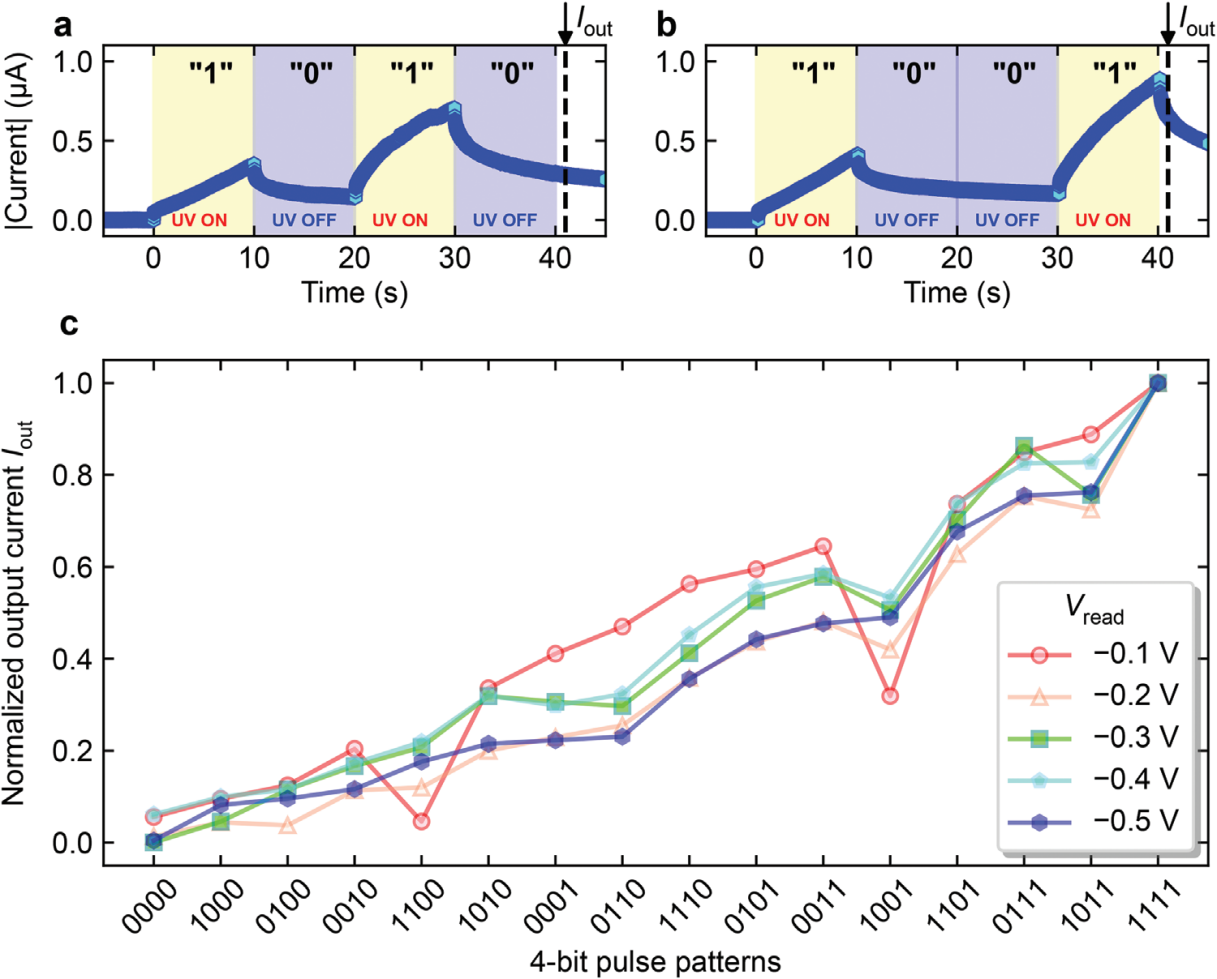
Output of the device when a 4‐bit encoded UV pulse is input to the ITO/Nb:STO junction. The output currents of the device for 4‐bit time‐series signals of a) “1010” and b) “1001” were input at *V*_read_ = −0.5 V. *I*_out_ is defined as the current value 1 s after completion of the input of the 4‐bit time‐series signals (dashed line in the figure). c) *I*_out_s for 16 patterns of 4‐bit time‐series signals normalized by *I*_out_ for the input of “1111.”

We next demonstrate that the tunability of the time constant exhibited by ITO/Nb:STO is suitable for processing time‐series signals. First, the output currents of the device for 4‐bit time‐series signals of “1010” and “1001” were input at *V*_read_ = −0.5 V, as shown in **Figures** **5**a and 5b, respectively. Here, the timescale of the time‐series signals was 10 s; UV ON = “1” and UV OFF = “0”; and the 4‐bit time‐series signals were written to the device using UV pulses; the output current (*I*_out_) is the current value 1 s after completion of the input of the 4‐bit time‐series signals as shown by the dashed line in Figure 5a,b. Consequently, *I*_out_ = 0.29 and 0.65 μA for “1010” and “1001,” respectively. From these results, it can be inferred that the device can reflect the history of the signal input.

Figure 5c shows the results of inputting such 4‐bit time‐series signals and measuring *I*_out_ for all 16 patterns of 4‐bit signals. Here, *I*_out_ was normalized by *I*_out_ for the input of “1111.” Thus, the changes in *I*_out_ as a function of the 4‐bit time‐series signals for each *V*_read_ can be compared in the same figure. Figure 5c shows that *I*_out_ tended to become larger as more “1”s were input later. All sixteen patterns of 4‐bit signals showed different values of *I*_out_, indicating that the 4‐bit signals input to the device could be distinguished by *I*_out_. See Supporting Information for the photo‐induced current characteristics of the device when 16 patterns of UV pulse sequences of 4‐bit signals were input to the device (Figures S11– S15, Supporting Information).

**Figure 6:**
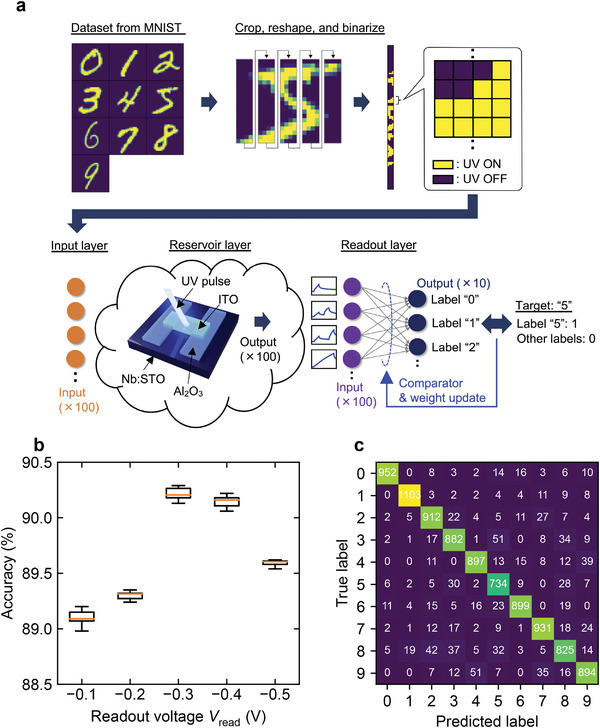
Handwritten digit image classification using a photonic memristor‐based PRC system. a) Process of reservoir calculation in this study. b) Estimated accuracy of handwritten digit image classification when ITO/Nb:STO junction was used as PR. c) Confusion matrix for the MNIST dataset (*V*_read_ = −0.3 V).

To evaluate the PR performance of the ITO/Nb:STO junction, a handwritten digit image classification task was performed using the Modified National Institute of Standards and Technology (MNIST) data set.^[^ ^39^ ^]^ **Figure** **6**a shows a schematic of the image classification task employed in this study (see Experimental Section for details of the pretreatment process and learning methods). Figure 6b shows the estimated classification accuracy. The highest accuracy (90.2%) was obtained at *V*_read_ = −0.3 V. This was considered to be due to the appropriate matching of the timescale of the time‐series signals and the relaxation time constant of the device. This implied that the learning could be optimized by selecting a *V*_read_ that showed an appropriate current relaxation time according to the timescale of the input signals. Figure 6c shows the confusion matrix for the MNIST dataset at *V*_read_ = −0.3 V. The misclassifications occurred mainly in identifying digits “5” or “8” as “3” and “4” or “7” as “9,” which are also difficult for human beings to distinguish at a resolution of 20 × 20 pixels. The misclassification trend was similar to that in a previous study.^[^ ^5^ ^]^ To clarify the contribution of the PR itself to the computational capability, we evaluated the accuracy of the system when the preprocessed data (an image with 100 × 4 pixels) were fed directly to the readout layer (without the dynamic reservoir), and the result was 85.1%. This comparison was made considering the same number of output neurons (i.e., only the last pixel was fed to the readout). Thus, the effectiveness of the proposed ITO/Nb:STO junction as a PR in improving the classification accuracy while reducing the computational cost was confirmed. Note that when the image with all 400 × 1 pixels was fed directly to a readout layer consisting of a single layer perceptron with 400 input nodes and 10 output nodes, the classification accuracy was 91.6%. This value was higher than that with PR (90.2%) as a result of the training with a larger network size. Compared to the network size of the proposed PRC system (100 × 10), the number of weights that could be optimized increased fourfold. This result was reasonable in terms of the RC concept of fast learning at a low computational cost, even at the expense of the computational performance. **Table** **2** lists the results of comparing the classification accuracy with that of the memristor‐based PRC system from previous studies.

**Table 2:** Comparison of the MNIST recognition results using memristor‐based PRC system.

Reservoir layer	Readout layer	Network size	Accuracy	Reference
SiO_*x*_‐based memristor^a)^	1T1R^c)^	220 × 10	83%^d)^	^[^ ^5^ ^]^
WO_*x*_‐based memristor^a)^	Software	88 × 10	85.6%^e)^	^[^ ^4^ ^]^
HfO_2_‐based memristor^a)^	Software	196 × 10	90%	^[^ ^8^ ^]^
SrTiO_3_‐based photonic memristor^b)^	Software	100 × 10	90.2%	Present study
Nanowire networks^a)^	TaO_*x*_‐based memristor	195 × 10	90.4%	^[^ ^40^ ^]^

^a)^ Full hardware implementation;

^b)^ Simulation‐assisted hardware implementation. Since a single memristor was used, the reservoir output was virtually constructed when high‐dimensional image data was input (see Experimental Section for details);

^c)^ One‐transistor one‐memristor;

^d)^ This results from in situ training;

^e)^ These results were validated on 14 000 (2000) training (test) datasets.

## 6 Conclusion

The photo‐induced current characteristics of the device under the UV irradiation were evaluated, and it was found that the timescale of the transient response of the device could be modulated by more than several orders of magnitude by varying the readout voltage (*V*_read_). This property suggested that a single device could process time‐series signals of various timescales that occur in living environments. An evaluation of the PR performance of the device in an image classification task showed that learning was optimized by selecting a *V*_read_ that exhibited an appropriate current relaxation time depending on the timescale of the input signals. Therefore, this device is expected to have a wide range of applications at the edge as an AI device for edge computing.

## 7 Experimental Section

### Device Fabrication

Commercially available Nb:STO (100) single‐crystal substrates (K&R Creation Co., Ltd.) of size 10 × 10 × 0.5 mm^3^ were used for the device fabrication; one side was mirror polished, and the Nb doping level was 0.5 wt%. To realize the crosspoint structure, Al_2_O_3_ film stripes with a thickness of 150 nm were applied to the polished surface of the substrate through magnetron sputtering (DC 300 W, 0.5 Pa). The reactive sputtering was performed with Ar/O_2_ gases flowing at a ratio of 90.0/30.0 sccm. Then, a rectangular ITO top EL with a thickness of 200 nm was deposited orthogonally to the stripes by magnetron sputtering (RF 60 W, 2.0 Pa), resulting in an ITO/Nb:STO junction device area of 200 × 200 µm^2^. Finally, Ti (100 nm) and then Pt (100 nm) were deposited by magnetron sputtering (RF 100 W, 2.0 Pa) to form a Pt/Ti EL. The low work function of Ti ensured ohmic contact with the n‐type semiconductor Nb:STO during electrical measurements and the Pt film prevented Ti oxidation. All the thin films were grown at room temperature.

### Device Evaluation

A source measure unit (Keysight B2901A) was used for all of the electrical measurements. The UV light was irradiated using an ultrahigh‐pressure mercury vapor lamp (Ushio Optical Module X) as the light source. The UV lamp provided several characteristic wavelengths in the range of 300–600 nm, including the wavelengths of 310, 370, and 440 nm. PL measurements were performed using a micro photoluminescence measurement system (HORIBA LabRAM‐HR PL). The excitation light source was an He‐Cd laser with a wavelength of 325 nm.

### Image Classification Task

The Modified National Institute of Standards and Technology (MNIST) database^[^ ^39^ ^]^ was used as the dataset. It is a large database of handwritten digit images and is commonly used for training and testing in the field of machine learning. This database consists of 60 000 training samples and 10 000 test samples. A 28 × 28 pixels original image provided by MNIST was cut into 20 × 20 pixels by deleting the edges not related to the numbers, divided into five columns, and sequentially joined together to form a 100 × 4 pixels image. Because the value of each pixel was represented by an 8‐bit integer ranging from 0 to 255, it was normalized to limit the value range from 0 to 1. Pixels with a value of 0.2 or higher were set to 1, and all other pixels were set to 0. See Supporting Information for the effect of the threshold used to binarize the input patterns on the accuracy of the system (Figure S16, Supporting Information). Then, a UV pulse train encoded in 4‐bit (“1” for UV ON, “0” for UV OFF) was generated. Each pulse train was input to the ITO/Nb:STO junction, and *I*_out_ was measured, for a total of 100 *I*_out_ data points as the reservoir output. Each time a different pattern was input, a voltage sweep (0 V → +1 V → 0 V → −1 V → 0 V) was performed to initialize the device state. In this study, the output of a preprocessed MNIST image input to the device was virtually constructed by substituting the stored *I*_out_ values into the corresponding 4‐bit columns. See Supporting Information for the reproducibility of *I*_out_ (Figure S17, Supporting Information). Then, the *I*_out_ data were input to a single‐layer perceptron with 100 input neurons and 10 output neurons. A softmax function was used as the activation function, and the final output was obtained by calculating the dot product of the *I*_out_ data and joint weights between the input and output neurons. To minimize cross‐entropy, learning was supervised based on an error backpropagation method using the RMSprop method.^[^ ^41^ ^]^ Similar methods were often used to evaluate the performance of PR with an image classification task using MNIST dataset.^[^ ^4^, ^5^, ^8^, ^14^, ^40^ ^]^ To evaluate the PR performance, 60 000 handwritten digit images were used from the MNIST training dataset, were iterated over 20 epochs with a batch size of 128, and then were validated using 10 000 handwritten digit images from the MNIST test dataset. This training and validation process was performed a total of ten times, and the estimated classification accuracy values were averaged to cancel out any variation in the training process.

## Conflict of Interest

The authors declare no conflict of interest.

## Supporting information

Supporting Information

## Data Availability

The data that support the findings of this study are available from the corresponding author upon reasonable request.
