# Supplementary material for: Photonic Physical Reservoir Computing with Tunable Relaxation Time Constant
Source: Adv Sci (Weinh). 2023 Nov 20;11(3):2304804. doi: 10.1002/advs.202304804 (PMC10797460; doi:10.1002/advs.202304804)
Supplement: Supplementary file 1 — Supporting Information [file ADVS-11-2304804-s001.pdf]

ADVANCED SCIENCE
Open Access

Supporting Information

for *Adv. Sci.*, DOI 10.1002/advs.202304804

Photonic Physical Reservoir Computing with Tunable Relaxation Time Constant

*Yutaro Yamazaki and Kentaro Kinoshita**

Photonic Physical Reservoir Computing with Tunable Relaxation Time Constant

*Yutaro Yamazaki and Kentaro Kinoshita**

Department of Applied Physics, Tokyo University of Science
6–3–1 Niijuku, Katsushika-ku, Tokyo,125–8585 Japan
Email Address: kkinosita@rs.tus.ac.jp

Figure S1 shows a schematic of the Sn-doped In_2O_3 (ITO)/Nb-doped $SrTiO_3$ (Nb:STO) junction with a structure to avoid the direct contact of the probe with the area of the ITO/Nb:STO interface. Adopting this structure made it possible to prevent light from being reflected by the measurement probe during light irradiation and ensured that the ultraviolet (UV) light was incident on the ITO/Nb:STO interface.

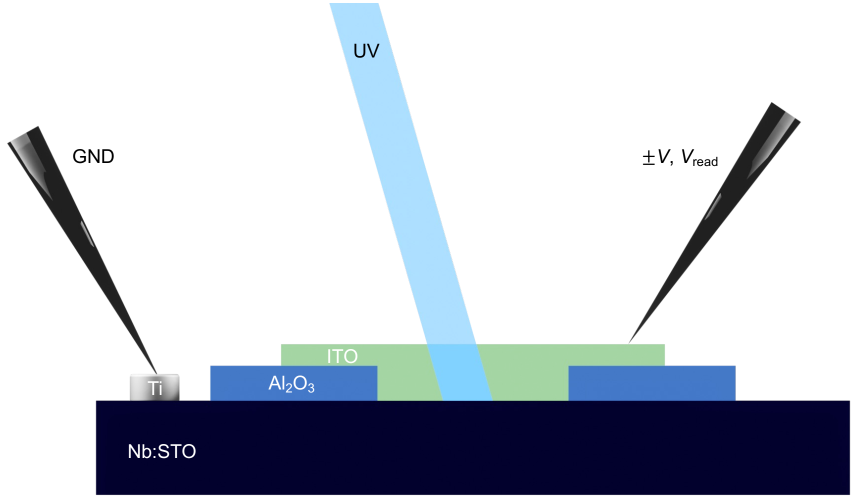

Figure S1. Schematic of ITO/Nb:STO junction (cross-sectional view).

Figure S2 shows cyclic endurance of the ITO/Nb:STO junction until 200 switching cycles, where the readout voltage (V_{read}) was +0.3 V. The transitions from a high-resistance state (HRS) to a low-resistance state (LRS) and vice versa are called SET and RESET, respectively. The one cycle consists of a set of SET and RESET switching. A memory window, the resistance ratio of HRS to LRS, was retained more than 10^3 through 200 switching cycles.

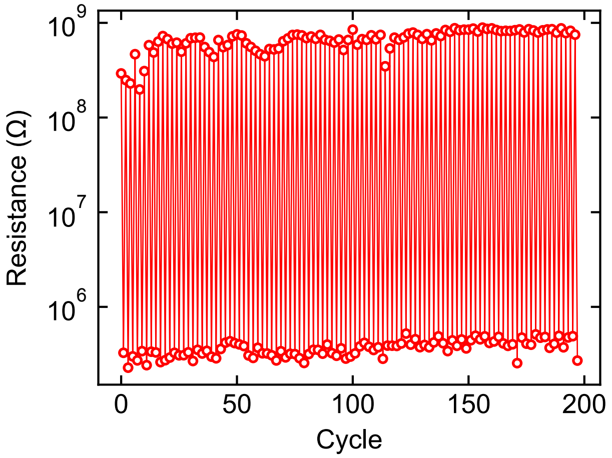

Figure S2. Cyclic endurance of the device.

Figure S3 shows the measured current retention characteristics after switching the device to LRS by applying a positive bias (+ 3 V). The V_{read} was +0.3 V. The current value gradually relaxes over approximately 10^3 s. This forgetting characteristic is similar to those observed in memristors with the structure of metal (Pt)/Nb:STO junction [1, 2]. In recent years, this property has been found to be suitable for neuromorphic computing [3, 4, 5].

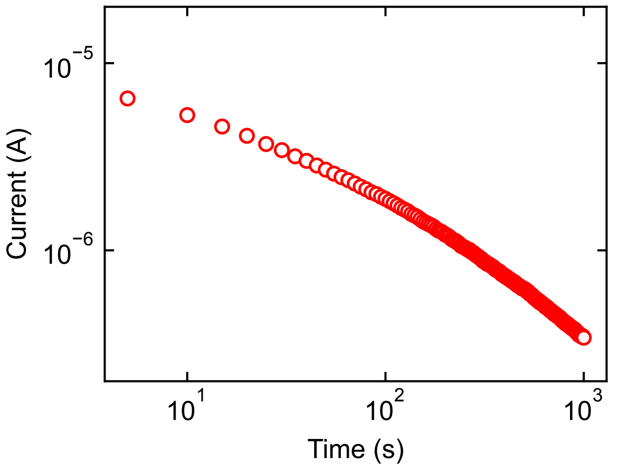

Figure S3. Current retention characteristics of the device.

Figure S4 shows the area dependence of the initial resistance of the ITO/Nb:STO junction device. The initial resistance (R) is inversely proportional to the device area (S), $R \propto 1/S$ (see **Figure S5**). This result suggests that the resistive switching takes place over the entire interface area. After the initial switching cycle, we continued to switch the device further and investigated the area dependence of resistances in HRS and LRS.

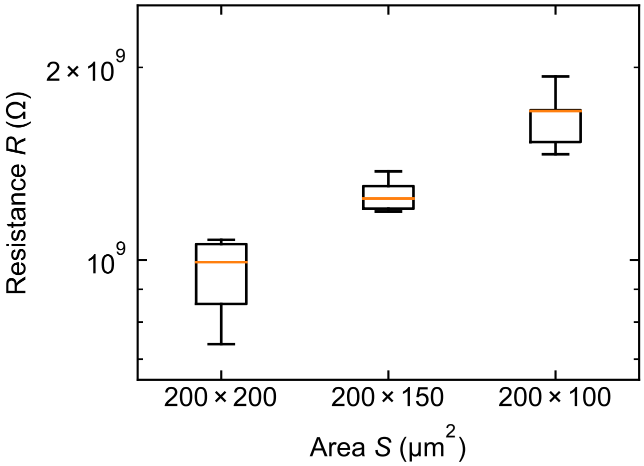

Figure S4. Device area dependence of initial resistance.

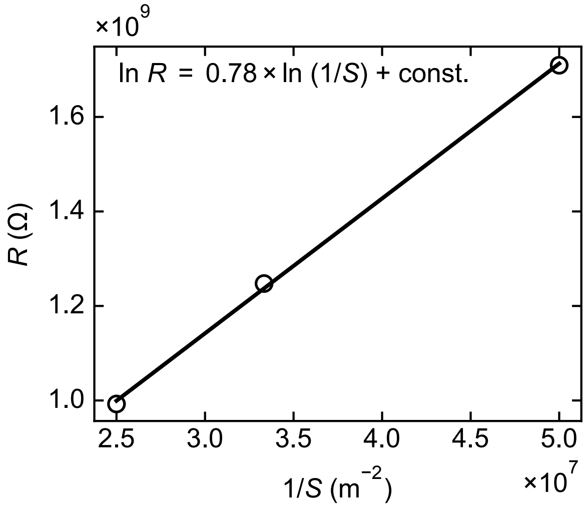

Figure S5. The relationship between resistance and the inverse of device area. The resistance value used for fitting was the median resistance value for each device area in Figure S4.

Figure S6 shows the area dependence of resistances in HRS and in LRS. The resistances in both HRS and LRS are inversely proportional to the device area, $R \propto 1/S$ (see **Figure S7** and **Figure S8**) similarly to that was confirmed for the initial resistance. Therefore, it was suggested that the resistive switching takes place over the entire interface area even after repeating resistance switching. This result, which was observed in, is similar to that of Pt/Nb:STO junction memristors reported by Sim et al [6], and consistent with the report that the resistive switching of memristors with the structure of metal (Pt, Ni)/Nb:STO junction occur in the entire interface area [6, 7, 8, 9].

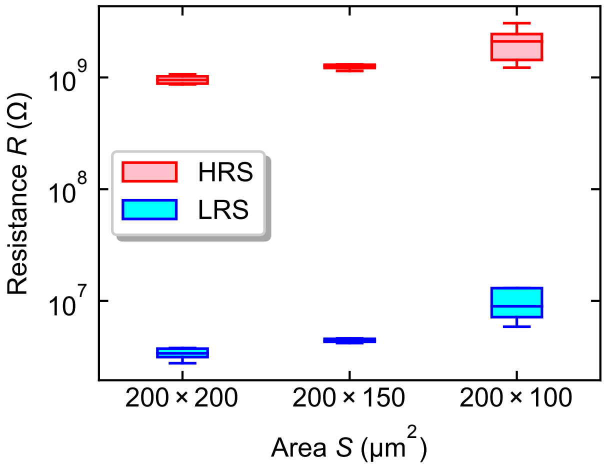

Figure S6. Device area dependence of resistance after switching.

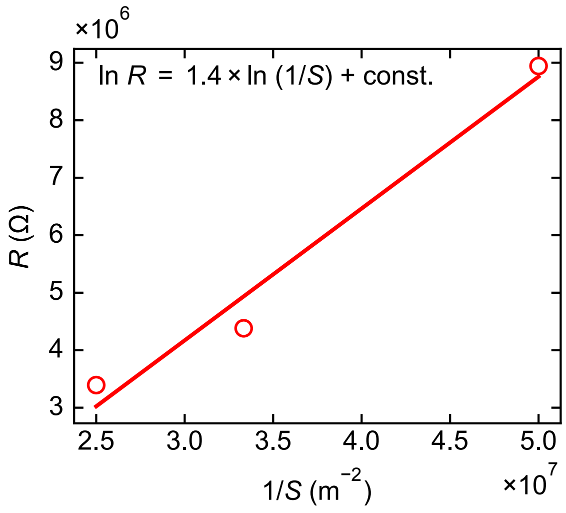

Figure S7. The relationship between resistance and the inverse of device area (HRS). The resistance value used for fitting was the median resistance value for each device area in Figure S6.

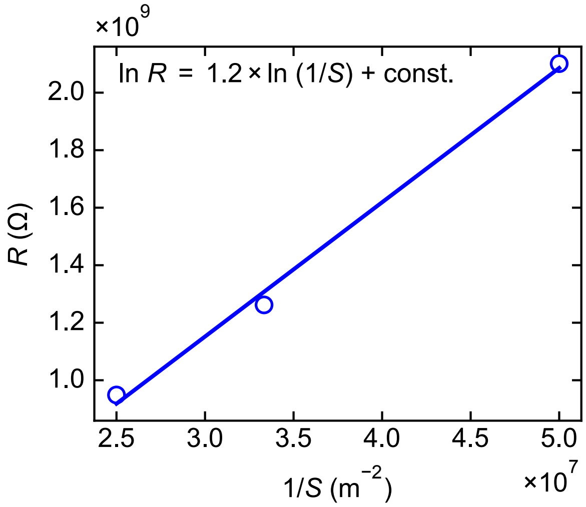

Figure S8. The relationship between resistance and the inverse of device area (LRS). The resistance value used for fitting was the median resistance value for each device area in Figure S6.

We performed peak fitting of the obtained photoluminescence (PL) spectra in order to discuss the recombination centers attributed to the luminescence. For all the PL spectra shown in **Figures 4a–c** in the main text, optimal fitting results were obtained by assuming the addition of the three Gaussian functions. The Gaussian used in the fitting is given by **Equation S1**,

$$I = \sum_{i=1}^{3} S_i \exp\left[-\frac{\left(E - E_i^{\mathrm{peak}}\right)^2}{\Gamma_i^2}\right] + A, \tag{S1}$$

where I is the intensity of the PL spectrum, S_i is the intensity parameter in each Gaussian, E is the energy, E_i^{peak} is the peak energy in each Gaussian, Γ_i is the broadening parameter in each Gaussian, and A is the background intensity. **Tables S1–S3** show the peak fitting results for Figures 4a–c in the main text, respectively, while A's were determined to be 81.6, 65.1, and 96.7, respectively.

Table S1. Peak fitting results in Figure 4a.

i	S_i (arb. units)	E_i^{peak} (eV)	Γ_i (eV)
1	1.25×10^{3}	2.81	4.04×10^{-1}
2	1.07×10^{3}	3.03	2.58×10^{-1}
3	5.78×10^{2}	3.21	1.11×10^{-1}

Table S2. Peak fitting results in Figure 4b.

i	S_i (arb. units)	E_i^{peak} (eV)	Γ_i (eV)
1	9.60×10^{2}	2.77	3.99×10^{-1}
2	1.18×10^{3}	3.01	2.63×10^{-1}
3	7.21×10^{2}	3.21	1.26×10^{-1}

Table S3. Peak fitting results in Figure 4c.

i	S_i (arb. units)	E_i^{peak} (eV)	Γ_i (eV)
1	1.59×10^{3}	2.89	4.22×10^{-1}
2	6.05×10^{2}	3.07	2.06×10^{-1}
3	4.85×10^{2}	3.22	8.22×10^{-2}

Figure S9 and **Figure S10** show the time dependence of the current (I–t characteristics) when +0.5 V or −0.5 V was applied to the device for 3000 s, respectively.

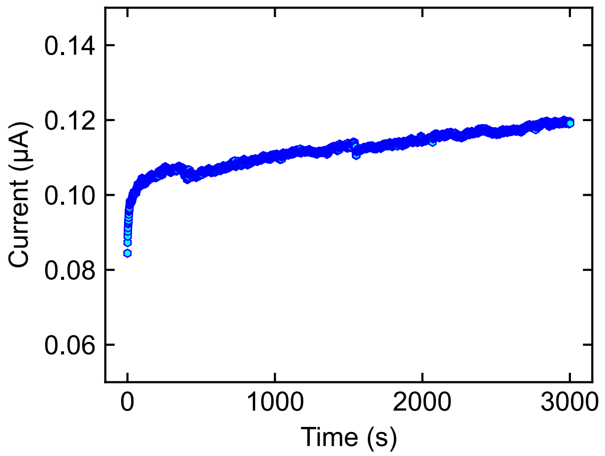

Figure S9. I–t characteristics (+0.5 V, 3000 s).

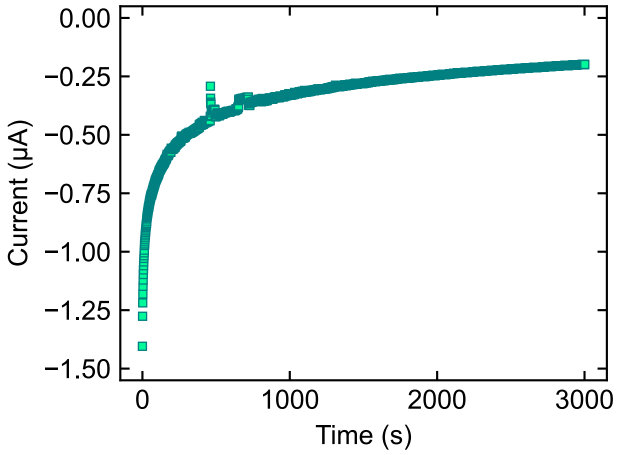

Figure S10. I–t characteristics (−0.5 V, 3000 s).

Figures S11–S15 show the photo-induced current characteristics of the device when irradiated with of UV pulse train of 4-bit signals of 16 patterns.

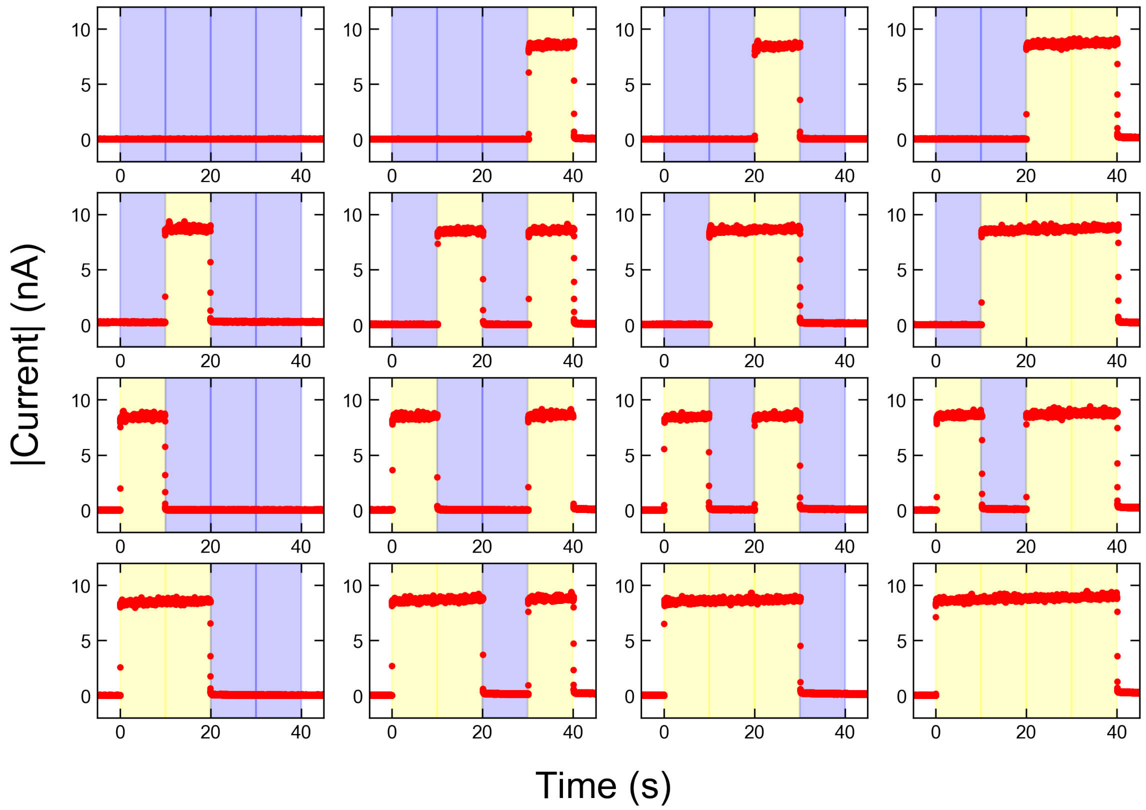

Figure S11. Dynamic photo-induced current responses to all combinations of 4-bit UV pulse patterns from “0000” (upper left) to “1111” (lower right) when $V_{\mathrm{read}} = -0.1$ V.

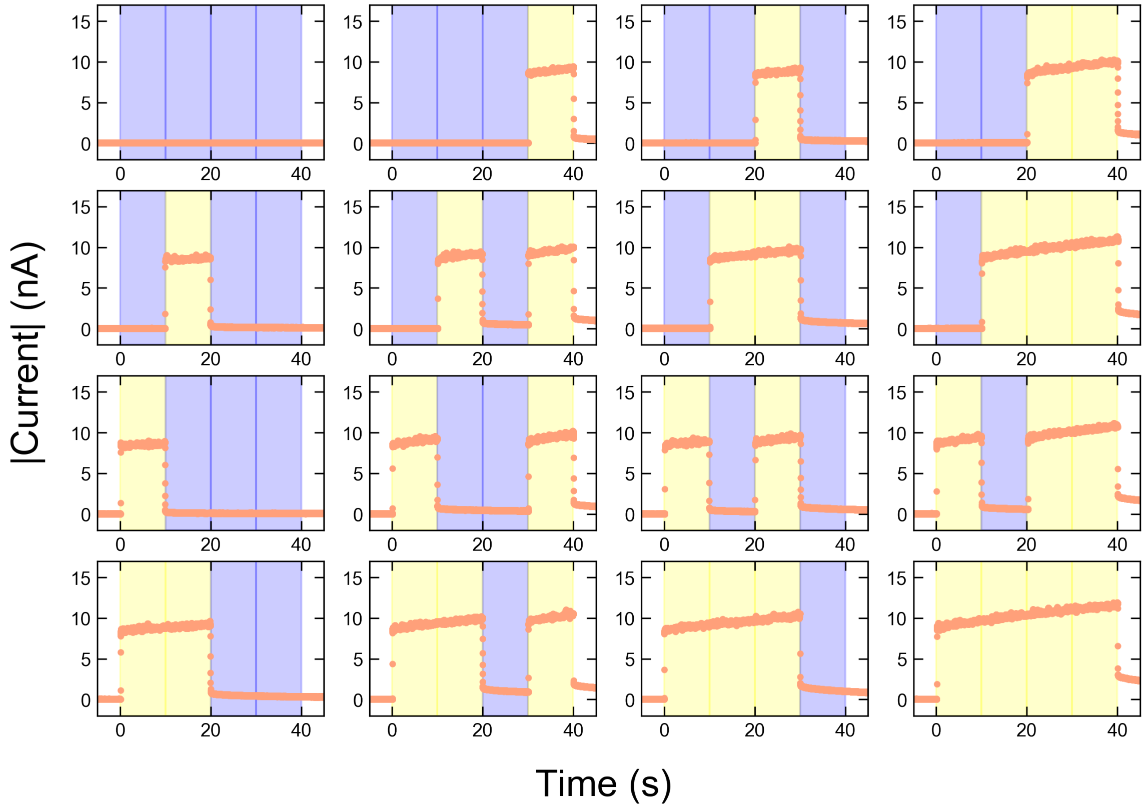

Figure S12. Dynamic photo-induced current responses to all combinations of 4-bit UV pulse patterns from “0000” (upper left) to “1111” (lower right) when $V_{\mathrm{read}} = -0.2$ V.

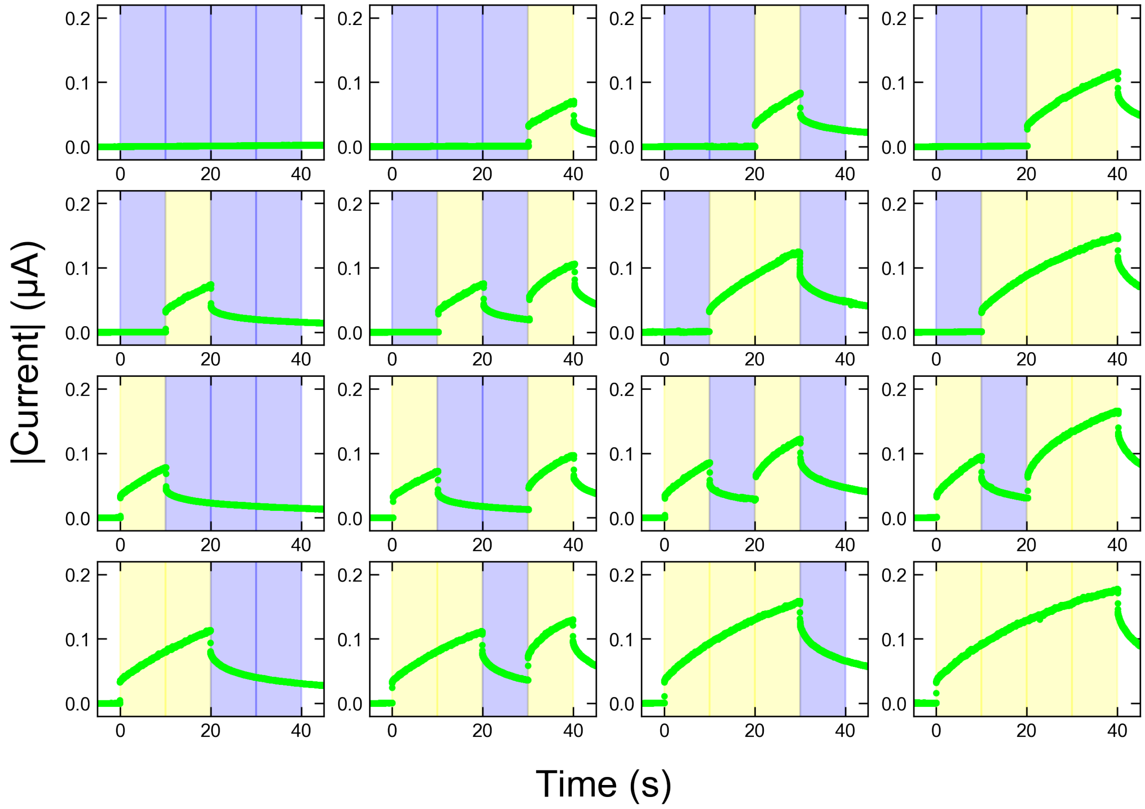

Figure S13. Dynamic photo-induced current responses to all combinations of 4-bit UV pulse patterns from “0000” (upper left) to “1111” (lower right) when $V_{\mathrm{read}} = -0.3$ V.

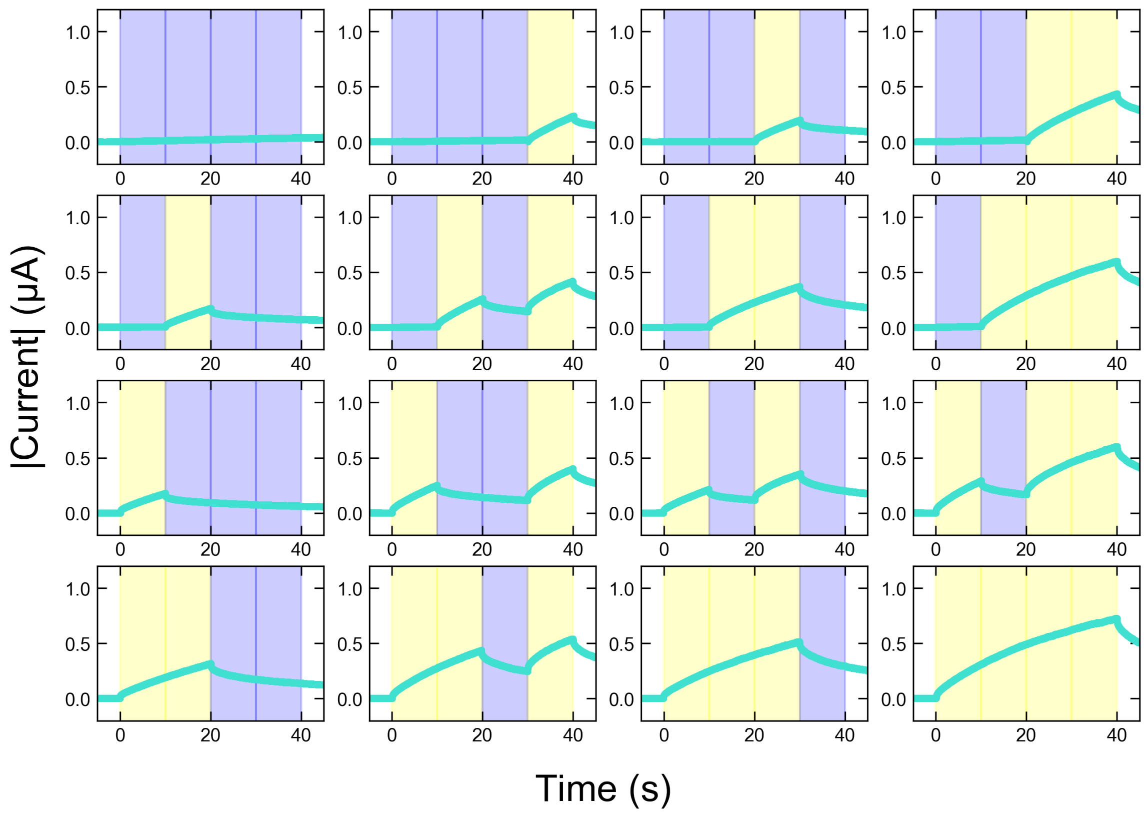

Figure S14. Dynamic photo-induced current responses to all combinations of 4-bit UV pulse patterns from "0000" (upper left) to "1111" (lower right) when $V_{\mathrm{read}} = -0.4$ V.

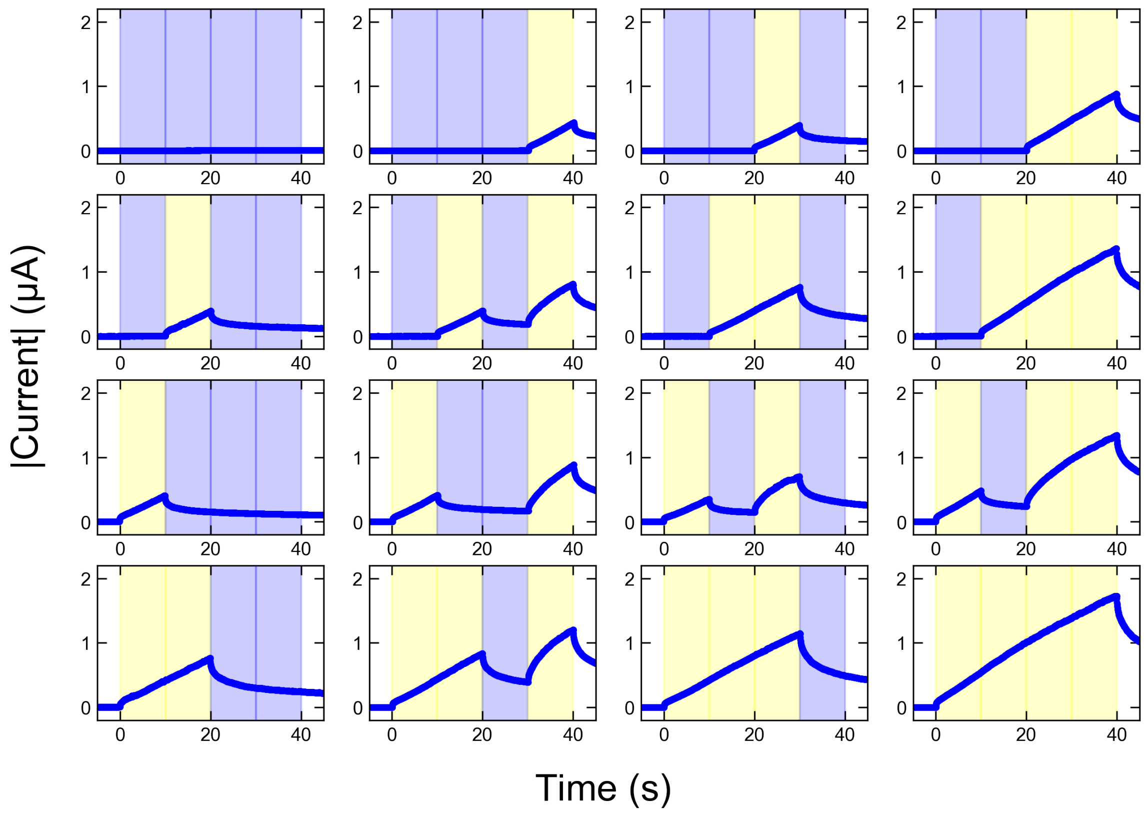

Figure S15. Dynamic photo-induced current responses to all combinations of 4-bit UV pulse patterns from "0000" (upper left) to "1111" (lower right) when $V_{\mathrm{read}} = -0.5$ V.

Figure S16 shows the results of an analysis of the effect of the threshold used to binarize the input patterns on the accuracy of the system.

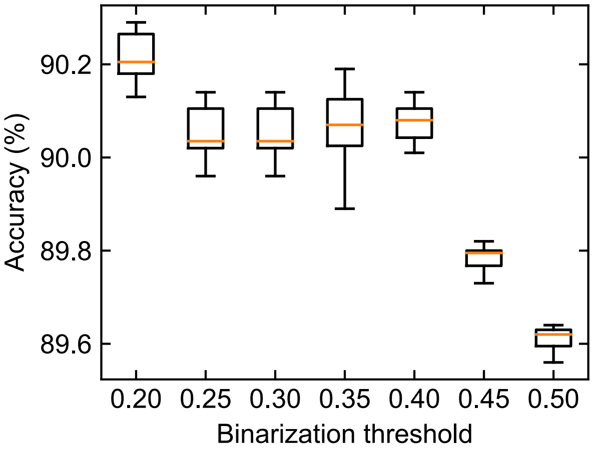

Figure S16. The effect of the threshold used to binarize the input patterns on the accuracy of the system (when $V_{\mathrm{read}} = -0.3$ V). This training and validation process was performed a total of 10 times, and the estimated classification accuracy was averaged to cancel out the variation in each training process.

Figure S17 shows the reproducibility of the output current (I_{out}) at $V_{\mathrm{read}} = -0.3$ V, which is the best performance shown in the main text. The measurement was repeated three times, the plotted points represent the average values, and the error bars represent the maximum and minimum values. The reproducibility is good for the same 4-bit pattern. On the other hand, the difference in I_{out} between "0110," "0001," and "1010" and between "1001" and "0101" is small. This tendency is undesirable in a task that requires accurate classification of 4-bit patterns. However, when processing time-series signals, this randomness can have a positive effect. The addition of another heterogeneity or randomness to the reservoir components improves performance, as shown for example in the following references [10, 11].

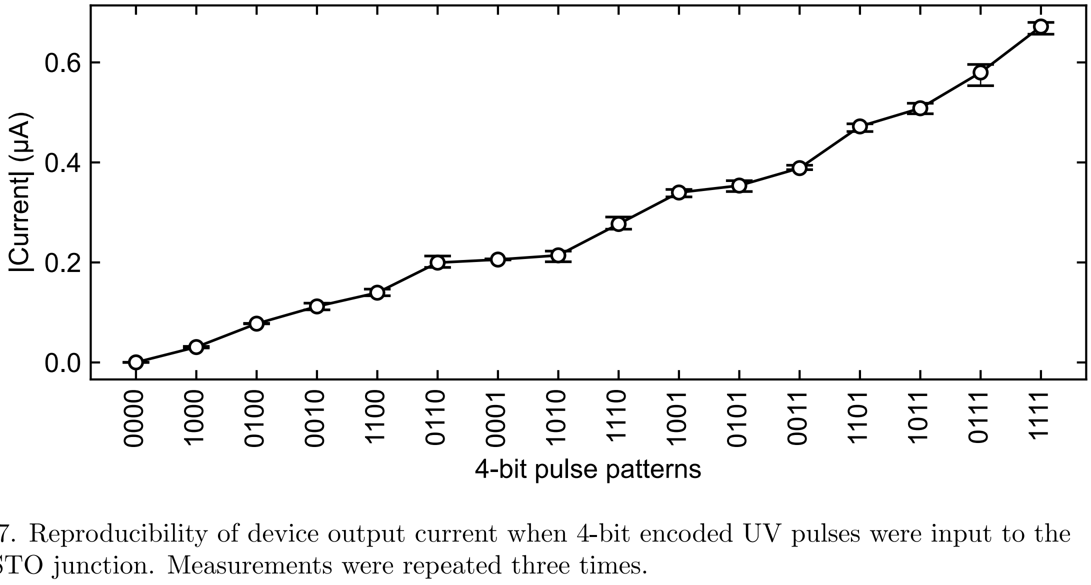

Figure S17. Reproducibility of device output current when 4-bit encoded UV pulses were input to the ITO/Nb:STO junction. Measurements were repeated three times.

References

[1] E. Mikheev, B. D. Hoskins, D. B. Strukov, S. Stemmer, *Nature Communications* **2014**, *5*, 1.

[2] E. M. Bourim, Y. Kim, D.-W. Kim, *ECS Journal of Solid State Science and Technology* **2014**, *3*, N95.

[3] Z.-H. Tan, X.-B. Yin, R. Yang, S.-B. Mi, C.-L. Jia, X. Guo, *Scientific Reports* **2017**, *7*, 713.

[4] S. Majumdar, H. Tan, I. Pande, S. Van Dijken, *APL Materials* **2019**, *7*, 091114.

[5] N. Yang, J. Zhang, J.-K. Huang, Y. Liu, J. Shi, Q. Si, J. Yang, S. Li, *ACS Applied Electronic Materials* **2022**, *4*, 3154.

[6] H. Sim, H. Choi, D. Lee, M. Chang, D. Choi, Y. Son, E.-H. Lee, W. Kim, Y. Park, I.-K. Yoo, et al., In *IEEE InternationalElectron Devices Meeting, 2005. IEDM Technical Digest.* IEEE, **2005**, 758.

[7] S. Lee, J. S. Lee, J.-B. Park, Y. Koo Kyoung, M.-J. Lee, T. Won Noh, *APL Materials* **2014**, *2*, 066103.

[8] J. Park, D.-H. Kwon, H. Park, C. Jung, M. Kim, *Applied Physics Letters* **2014**, *105*, 183103.

[9] X.-B. Yin, Z.-H. Tan, X. Guo, *Physical Chemistry Chemical Physics* **2015**, *17*, 134.

[10] G. Tanaka, R. Nakane, T. Yamane, D. Nakano, S. Takeda, S. Nakagawa, A. Hirose, In *Neural Information Processing*. Springer International Publishing, Cham, **2016**, 187.

[11] G. Tanaka, T. Matsumori, H. Yoshida, K. Aihara, *Physical Review Research* **2022**, *4*, L032014.
